# Inflammation in the pathogenesis of microvascular complications in diabetes

**DOI:** 10.3389/fendo.2012.00170

**Published:** 2012-12-21

**Authors:** Dung V. Nguyen, Lynn C. Shaw, Maria B. Grant

**Affiliations:** Department of Pharmacology and Therapeutics, University of Florida, College of MedicineGainesville, FL, USA

**Keywords:** inflammation, diabetes mellitus, microvascular complications, oxidative stress, advanced glycation end-products, inflammatory cytokines

## Abstract

Diabetes and hyperglycemia create a proinflammatory microenvironment that progresses to microvascular complications such as nephropathy, retinopathy, and neuropathy. Diet-induced insulin resistance is a potential initiator of this change in type 2 diabetes which can increase adipokines and generate a chronic low-grade inflammatory state. Advanced glycation end-products and its receptor, glycation end-products AGE receptor axis, reactive oxygen species, and hypoxia can also interact to worsen complications. Numerous efforts have gained way to understanding the mechanisms of these modulators and attenuation of the inflammatory response, however, effective treatments have still not emerged. The complexity of inflammatory signaling may suggest a need for multi-targeted therapy. This review presents recent findings aimed at new treatment strategies.

## INTRODUCTION

**FIGURE 1:**
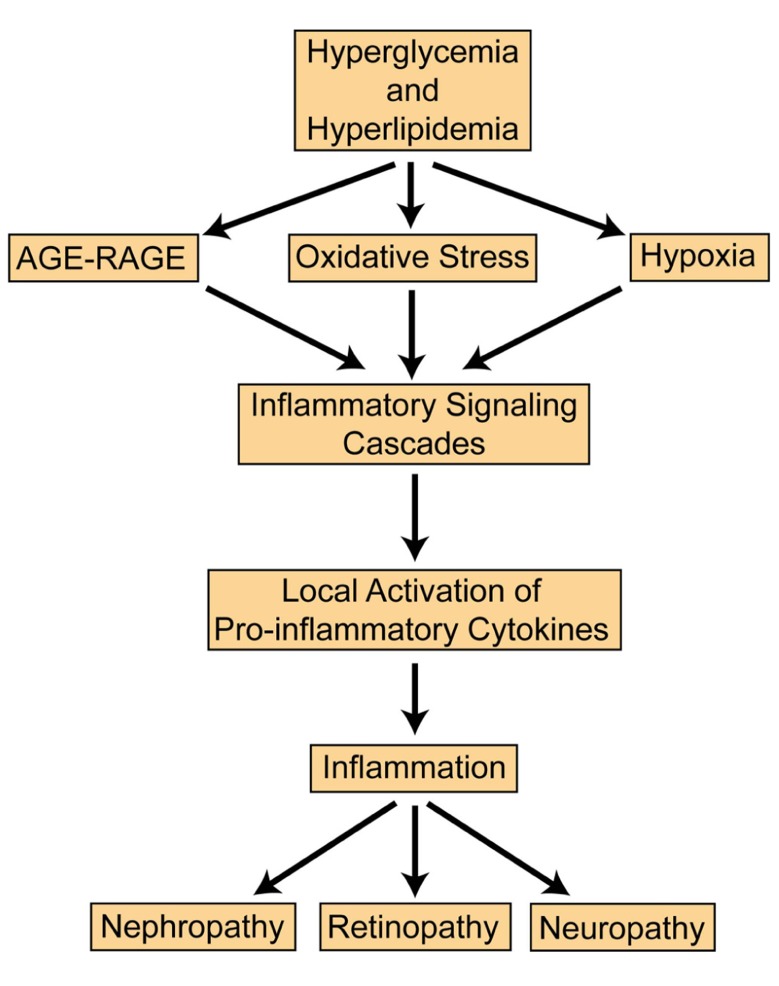
**General pathway in the progression of diabetic microvascular complications**.

Inflammation plays an essential role in the progression of diabetic microvascular complications. Proinflammatory cytokines C-reactive protein, tumor necrosis factor (TNF)-α, and interleukin (IL)-6 all demonstrate increased expression in diabetes (Peters et al., 1986; Ford, 1999; Festa et al., 2000; Müller et al., 2002; Temelkova-Kurktschiev et al., 2002). In chronic hyperglycemia, cytokines infiltrate vascular tissues and inhibit function and repair. Obesity is a major risk factor for diabetes and can induce inflammation by Toll-like receptor (TLR) activation to recruit proinflammatory cytokines and chemokines (Kwon et al., 2012). With the onset of diabetes, adipokines such as TNF-α and IL-6 may contribute to insulin resistance (Rajala and Scherer, 2003; Suganami et al., 2005). Adiponectin is initially upregulated to increase glucose uptake, and nitric oxide (NO) production; however, continued obesity may reduce adiponectin leading to complications observed in type 2 diabetes (T2D; Berg et al., 2001; Matsuzawa, 2005). Obesity is also associated with hyperlipidemia with elevated levels of cholesterol and triglycerides which may contribute to inflammation and diabetic retinopathy (DR; Dodson et al., 1981). The Fenofibrate Intervention and Event Lowering in Diabetes (FIELD) study found no relationship between serum lipid levels and DR (Keech et al., 2007; Chew et al., 2010). Fenofibrate is known to lower lipid levels, but it can also activate peroxisome proliferator-activated receptors (PPARs) and suppress inflammation by inhibiting nuclear factor kappa B (NF-κB; Tomizawa et al., 2011). As metabolic syndrome and inflammation persist, oxidative stress, hypoxia, and advanced glycation end-products (AGEs)/AGE receptor (RAGE) converge to exacerbate the problem (Brownlee, 2005; Vincent et al., 2011). A schematic summarizing the pathogenesis of diabetic microvascular complications is presented (**Figure 1**). The focus of this review is to overview the most recent findings relevant to treating nephropathy, retinopathy, and neuropathy.

## DIABETIC NEPHROPATHY

Diabetic nephropathy (DN) is the leading cause of end-stage renal disease (Nilsson et al., 2008). DN results in basement membrane thickening, expansion of the mesangium, reduced filtration, albuminuria, and ultimately renal failure (Graves and Kayal, 2008). Inflammatory cells can accumulate in glomeruli and interstitium to worsen DN (Lim and Tesch, 2012). Recent findings have identified a few key receptors involved in renal protection. Studies targeting these pathways along with other known mediators of inflammation have revealed the importance of inflammation in worsening DN.

Peroxisome proliferator-activated receptors are activated in response to fatty acids and regulate lipid and glucose homeostasis (Wahli and Michalik, 2012). In the kidney, PPARγ expression has been found in medullary collecting ducts, pelvic urothelium, and isolated glomeruli and cultured mesangial cells (Iwashima et al., 1999; Yang et al., 1999; Asano et al., 2000; Kume et al., 2008). Pioglitazone, a PPARγ agonist, increased anti-oxidant activity and reduced inflammation in hyperoxaluric rats (Taguchi et al., 2012). This suggests activation of PPARγ may have renoprotective functions. Similarly, the same agonist treated in T2D diabetic rats showed improved insulin resistance, glycemic control, and lipid profile while reducing inflammation by reducing macrophage infiltration and NF-κB expression (Ko et al., 2008).

Resveratrol (*trans*-3,4^′^,5-trihydroxyestilbene, RSV) is a polyphenolic compound found in grapes and other plants providing anti-oxidant effects (Chang et al., 2011). RSV improved renal function and reduced oxidative stress in type 1 diabetic (T1D) rats (Sharma et al., 2006; Dhaunsi and Bitar, 2012). Similarly, RSV treatment showed significant decreases in superoxide anion and protein carbonyl oxidative stress markers (Chang et al., 2011). RSV was shown to reduce renal lipotoxicity and mesangial cell glucotoxicity in diabetic mice mediated through activation of PPARγ co-activator 1α (Kim et al., 2012). In another study, RSV reduced IL-1β in streptozotocin (STZ)-diabetic rat kidneys, but there was a significant increase in TNF-α and IL-6 levels independent of NF-κB activation, suggesting RSV has both stimulatory and inhibitory effects on cytokines simultaneously and achieving the optimal dose may be critical to establishing efficacy (Chang et al., 2011).

Fcγ receptors (FcγR) are present in leukocytes, glomerular, and mesangial cells (Gómez-Guerrero et al., 1994, 2002; Radeke et al., 2002). FcγR can bind to immunoglobulin G (IgG). Circulating oxidized LDL-containing immune complexes (oxLDL-IC) are increased in diabetes stimulate synthesis of IgGs in addition to other proinflammatory cytokines such as IL-1β, IL-6, IL-18, and TNF-α in Mono Mac 6 cells and primary human macrophages (Saad et al., 2006; Abdelsamie et al., 2011). Increased oxLDL-ICs also increases matrix production in mesenchymal mesangial cells through activation of FcγRI and FcγRIII to increase collagen IV production in nephropathy (Abdelsamie et al., 2011). Attenuating FcγR activity may reduce the development of a proinflammatory environment and enhanced matrix production. A genetic defect in FcγR attenuated diabetic renal injury based on histological analyses and reduced leukocyte accumulation in glomeruli and interstitium (Lopez-Parra et al., 2012). There was also a reduction in intracellular superoxide generation *in vivo* and oxidative response to oxLDL-ICs *in vitro* (Lopez-Parra et al., 2012).

Dietary lipids preceding diabetes have been shown to upregulate proinflammatory cytokines and TLR transcriptional levels along with downregulation of transcripts involved in glucose metabolism in epididymal and mesenteric white adipose tissue (Kwon et al., 2012). TLR are innate immune receptors that have been implicated in T1D, T2D, and its associated complications (de Kleijn and Pasterkamp, 2003; Park et al., 2004; Rudofsky et al., 2004; Wen et al., 2004; Lang et al., 2005). In DN, TLR4 expression was increased in T2D and uremic patients and in mouse mesangial cells, suggesting its role in monocyte recruitment (Kaur et al., 2012; Yang et al., 2012). Studies confirmed increased TLR4 activation when cells were incubated with high glucose (Kaur et al., 2012). Monocytes displaying CD14^+^CD16^+^surface markers in the kidney can associate with TLR and activate NF-κB, and STAT expression to further promote a proinflammatory microenvironment (Yang et al., 2012). Therapeutic targets correcting dysregulated TLR signaling may therefore be an important target against inflammation and complications within the kidney.

Advanced glycation end-product production is widely associated with diabetic microvascular complications. Recent studies showed little benefit using benfotiamine, a lipophilic thiamine-derivative that activates transketolase to reduce AGE precursors (Babaei-Jadidi et al., 2003; Karachalias et al., 2010). Benfotiamine had no effect in decreasing existing plasma AGE or increasing AGE excretion (Alkhalaf et al., 2012). Similarly, evaluation of benfotiamine in cerebral cortex of STZ-induced diabetic rats showed little effect on reducing AGEs and TNF-α, however, it slightly attenuated oxidative stress (Wu and Ren, 2006). Despite the outcome, this approach remains active and a recent proposal has aimed at modifying the delivery to have dual targets instead of singular targeting. Using a nanoparticle shell, both AGE and RAGE inhibitors will be encased within the shell to suppress both axes and redundancy not addressed with a single therapy (Zhou et al., 2012). The exterior of the shell will contain RAGE analogs, which can also provide specificity to AGEs and delivery of therapeutics (Zhou et al., 2012). This dual therapy approach is still in its infancy, but it may have potential benefits if pursued to target both receptors and its ligands.

Current standard treatment of DN targets the renin–angiotensin system (RAS) through usage of angiotensin converting enzyme (ACE) inhibitors to limit systemic blood pressure to control intraglomerular pressure (Bonegio and Susztak, 2012). Upstream targeting may further decrease RAS activity. Aliskiren, a direct renin inhibitor, has been recently evaluated in DN. Treatment using aliskiren showed a significant reduction in TNF-α and transforming growth factor (TGF)-β (Gandhi et al., 2012). Some studies have shown that TGF-β may have a role in influencing renal growth and inflammation as well as fibrosis and renal dysfunction (Ziyadeh et al., 2000; Phillips and Steadman, 2002).

## DIABETIC RETINOPATHY

Diabetic retinopathy is one of the leading causes of blindness in adults of working age adults. Background DR is characterized by ischemic injury which creates a hypoxic environment in ocular tissues. Hypoxia has been shown to induce microglia activation and recruitment to ischemic sites in retinas (Kielczewski et al., 2011). Vascular injury in background DR and proliferative DR (PDR) increases proinflammatory cytokines which can promote leukostasis and vascular endothelial growth factor (VEGF) mediated permeability in the retinal vasculature (Chistiakov, 2011).

The retinal pigment epithelium (RPE) provides functional barriers for the exchange of nutrients to photoreceptor cells. Under hyperglycemia, microglia and macrophages accumulate in the RPE in Goto Kakizaki rats (Omri et al., 2011). Increases in transepithelial pores compromise tight junction integrity and allow materials to enter the choroidal space (Omri et al., 2011). Presence of inflammation can reduce transepithelial resistance (TER) and impact ion gradient generation between membrane transporters and tight junctions (Rizzolo et al., 2011). TNF-α exposure to human RPE cells showed decreased TER (Peng et al., 2012). GPR109A is a G protein-coupled receptor (GPCR) present in RPE that is upregulated in diabetic mouse and human retina (Gambhir et al., 2012). GPR109A has immunomodulatory effects in adipose tissue and progression of atherosclerosis (Digby et al., 2010; Montecucco et al., 2010; Lukasova et al., 2011). Two ligands of GPR109A, niacin and β-hydroxybutyrate, was shown to suppress IL-6 and chemokine ligand-2 (CCL2) induced by TNF-α (Gambhir et al., 2012). Additional studies should explore potential value of modulating GPR109A activity with its ligands to suppress inflammation in the retina of those discussed as well as other proinflammatory cytokines (Gambhir et al., 2012).

β-catenin is a downstream effector of the Wnt pathway and is found to be increased in several diabetic rodent models and in humans (Chen et al., 2009). Increased β-catenin may be due to sustained Wnt signaling where it can also activate NF-κB to induce inflammation (Dale, 1998; Yamashina et al., 2006; Yan et al., 2008). DR is characterized by hypoxia and oxidative stress, which contribute to Wnt activation. Blockage of Wnt led to reduced inflammation through decreased ICAM-1 in the retina (Chen et al., 2009). Mab2F1, a monoclonal antibody targeting Wnt co-receptor LDL receptor-related protein 6 resulted in reduced retinal vascular leakage, inflammation, and attenuation of leukostasis (Lee et al., 2012).

Comparing cytokine levels of peripheral blood in diabetic patients revealed that levels of IL-22 expressed by T-helper (Th) 22 was significantly increased compared to controls, however, the differences were not significant between NPDR, PDR, and in diabetic patients without DR (Chen et al., 2012). IL-22 levels were also positively correlated with duration of diabetes (Chen et al., 2012). TNF-α has been shown to be increased in serum of diabetic patients. The results from this study suggest a potential role of Th22 expressing IL-22 levels in the pathogenesis of diabetic complications.

Increased RAGE levels and its ligand S100B are found in rat diabetic retinas and also found in cultured Müller glial cells exposed to high glucose (Limb et al., 2002; Zong et al., 2010). S100B has been shown induce inflammatory cytokines such as TNF-α and vascular CAM (VCAM)-1 in human microvascular endothelial cells (Valencia et al., 2004). Similarly, Müller glial cells treated with exogenous S100B showed increased levels of TNF-α, IL-6, IL-8, VEGF, and CCL2 (Zong et al., 2010). Treatment of S100B in cells showed a dose-dependent activation of mitogen-activated protein kinase pathway (MAPK) (Zong et al., 2010). *In vivo* studies should assess the relevant concentrations of S100B in pathogenesis of DR.

The RAS plays a vital role in regulating many physiological processes of the vascular system. Elevated levels of renin, prorenin, and Angiotensin II (Ang II) are found in patients with DR (Wilkinson-Berka, 2008). In PDR, prorenin and its receptor [(P)RR] are upregulated in retinal endothelial cells (Kanda et al., 2012). Increased (P)RR, prorenin, and activated prorenin were found in human vitreous fluid which can promote inflammatory angiogenesis in the eye (Satofuka et al., 2008; Kanda et al., 2012). (P)RR can activate extracellular signal-regulated kinases (ERK) and induce inflammatory responses in the eye (Kanda et al., 2012). Blockage of (P)RR reduced ERK activity and decreased diabetes-induced retinal inflammation (Satofuka et al., 2012).

**FIGURE 2:**
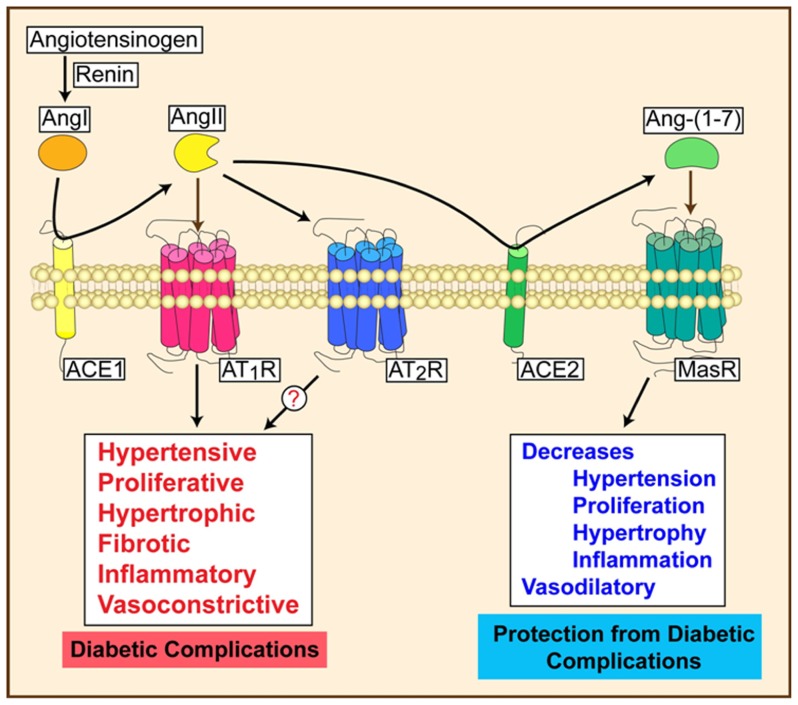
**Activation of RAS can lead to progressive or protective effects depending on the signaling mechanisms**.

Downstream effectors also have important functions in DR. Ang II, a product of ACE, activates the AT_1_ receptor to induce vasoconstriction, proliferation, fibrosis, and inflammation. The protective arm of the RAS involves ACE2, which produces Ang-(1-7). As a vasodilator peptide with anti-hypertensive, anti-hypertrophic, anti-fibrotic, and anti-thrombotic functions (3), Ang-(1-7) stimulates NO production by activating endothelial NO synthase (eNOS) in an Akt-dependent manner and decreases ROS production by attenuating NADPH oxidase. Ang-(1-7) mediates its effects by activating the GPCR, the Mas receptor (Sampaio et al., 2007; Benter et al., 2008). Chronic Ang-(1-7) treatment preserves endothelial function in rat models of myocardial ischemia and in-stent restenosis (Loot et al., 2002; Langeveld et al., 2005). Treatment with ACE2 or Ang-(1-7) corrected diabetic defects in therapeutic angiogenesis (Oudit et al., 2010; **Figure 2**). Intraocular administration of adeno-associated virus expressing ACE2/Ang-(1-7) significantly reduced CD45^+^ macrophages, CD11b^+^ microglial cells, and oxidative damage in mice (Verma et al., 2012). Targeting both upstream and downstream components of the RAS axis may provide synergistic effects in treating microvascular complications.

## DIABETIC NEUROPATHY

Diabetic neuropathy (DNO) is the most common complication of diabetes, where population-based studies have indicated more than half of the patients with either T1D or T2D develop DNO, and as much as 30% of those manifestations are painful (Harati, 2007; Ramos et al., 2007; Farmer et al., 2012). Recent reviews have emphasized the importance of targeting oxidative stress and inflammation in the treatment of DNO (Vincent et al., 2011; Farmer et al., 2012).

Tumor necrosis factor-α has been implicated in contributing to insulin resistance in obesity due to its increased expression in adipose tissue. Obese mice with a TNF-α^-^^/^^-^mutation displayed improved insulin sensitivity and lowered circulating fatty acids, improving obesity-induced glucose tolerance (Uysal et al., 1997). Increased plasma TNF-α and macrophages are also associated with the progression of DNO, suggesting continued expression of these cytokines contribute to diabetic microvascular complications (Purwata, 2011). Similar experiments evaluating TNF-α null mice showed that they are less susceptible to developing diabetic complications (Gao et al., 2007). Targeting TNF-α through pharmacological means can potentially reverse the deleterious effects in DNO. Infliximab, a monoclonal anti-TNF-α antibody approved for treatment of autoimmune diseases such as rheumatoid arthritis and psoriasis has been explored (Lin et al., 2008). Administration of infliximab into T1D mice showed significant improvement in neural function comparable to non-diabetic controls (Yamakawa et al., 2011).

Tumor necrosis factor-α can also influence AGE/RAGE activity making it a relevant target in DNO. In the progression of DNO, RAGE expression was increased in diabetic peripheral nerves and dorsal root ganglia (DRG; Toth et al., 2008). Mice models deficient in RAGE attenuated the structural and electrophysiological changes in peripheral nerves and DRG after prolonged diabetes of 5 months and also reduced NF-κB and protein kinase C activation (Toth et al., 2008). NF-κB can induce apoptosis, cell cycle, and plasticity, neurogenesis, and differentiation in the central nervous system (Foehr et al., 2000; Kumar et al., 2004; Fraser, 2006). RSV has been shown to inhibit NF-κB activity and TNF-α, IL-6, and cyclooxygenase-2 levels (Kumar and Sharma, 2010). BAY 11-7082, an inhibitor of kappa B (IκB) phosphorylation, downregulated NF-κB and led to improved sensory response, motor nerve conduction velocity, and nerve blood flow (Kumar et al., 2012). Similarly, there was a significant reduction in the oxidative stress marker, malondialdehyde, IL-6, and TNF-α levels (Kumar et al., 2012). While IL-6 is generally regarded as proinflammatory, its role in DNO is still unclear since IL-6 administration may have neurotrophic effects (Cotter et al., 2010).

Bradykinin B1 receptor (B1R) of the kallikrein–kinin system has been shown to be upregulated in response to increases of oxidative stress in diabetes (Dias et al., 2010). In another study, minocycline has been shown to exhibit anti-inflammatory and anti-oxidant effects by inhibiting microglia activation (Pabreja et al., 2011). Inhibition of microglia activation in STZ-diabetic rats using either fluorocitrate or minocycline reduced B1R expression along with IL-1β and TNF-α proinflammatory cytokines in spinal dorsal horn (Talbot et al., 2010). Microglia inhibitors may have an effect on thermal hyperalgesia and allodynia which support a role of B1R in pain neuropathy (Talbot et al., 2010). Antagonists to B1R showed a reversal of allodynia in STZ-diabetic rats, suggesting the mediation of early DNO due to inflammation (Talbot et al., 2010). However, in Akita mice, loss of B1R and bradykinin B2 receptor (B2R) appears to exacerbate nephropathy and neuropathy, suggesting that its activation in this diabetes model may be protective (Kakoki et al., 2010). Further studies should assess the role of B1R in different animal models of diabetes.

Angiopoietin-1 (Ang-1) has been demonstrated to have benefits against vascular leakage and endothelial cell survival (Cho et al., 2004). Variants have been developed to improve on solubility and potency (Cho et al., 2004). Matrilin-1-Ang-1 (MAT-Ang-1) has been demonstrated to have anti-inflammatory protection against cytokines IL-1α, IL-1β, IL-6, and TNF-α in sepsis (Alfieri et al., 2012). Another variant, cartilage oligomeric matrix protein (COMP)-Ang-1, has been hypothesized to improve regeneration of nerve fibers and endoneural microvessels in leptin-deficient obese (ob/ob) mice, a model for T2D (Kosacka et al., 2012). COMP-Ang-1 treatment was capable of reducing macrophage infiltration and T-cell number in sciatic nerves of ob/ob mice by 45 and 47%, respectively (Kosacka et al., 2012). Upstream effectors of Ang-1 have also recently been explored. Thymosin β4 improved diabetes-induced vascular dysfunction in sciatic nerve, nerve function and can mediate this through upregulation of Ang-1 in diabetic mice (Wang et al., 2012). Regulators of Ang-1 may therefore have benefits against neural and vascular dysfunction.

## CONCLUSION

The worldwide increase in prevalence of obesity and diet-induced insulin resistance increases the need to reduce chronic inflammation. Diabetic microvascular complications progress due to inflammation which originates from multiple pathways and mechanisms. This complexity warrants the need for effective therapies that target more than one signaling cascade. Inhibition of both inflammatory cytokines and their activators/regulators may provide additional coverage to treating nephropathy, retinopathy, and neuropathy. Similarly, this can be combined and optimized with anti-oxidant and AGE/RAGE therapies to mitigate compensatory mechanisms. As further studies emerge to address current limitations, improved therapies targeting diabetic microvascular complications may ultimately transition from treating the pathology to prevention.

## Conflict of Interest Statement

The authors declare that the research was conducted in the absence of any commercial or financial relationships that could be construed as a potential conflict of interest.
